# Editorial: Application of Optical Coherence Tomography Angiography in Retinal and Optic Nerve Disorders

**DOI:** 10.3389/fneur.2021.777156

**Published:** 2021-10-29

**Authors:** Christine Wen Leng Yau, Shaun Sebastian Sim, Chui Ming Gemmy Cheung

**Affiliations:** Singapore National Eye Centre, Singapore Eye Research Institute and Duke-NUS Graduate Medical School, Ophthalmology and Visual Sciences Academic Clinical Program, Singapore, Singapore

**Keywords:** retinal microvascular alterations, neuron, biomarker, image processing, diagnosis, monitoring

Optical coherence tomography angiography (OCTA) is a non-invasive imaging modality capable of directly assessing retinal microvascular changes without intravenous dye injection (1). It maps the movement of red blood cells through the degree of motion contrast, which corresponds to angiographic flow (2).

While fluorescein angiography (FA) has been widely used in clinical practice over the last 50 years to image flow, it has several limitations. One major limitation is that the visualization of retinal vessels is largely limited to the superficial vasculature (3). This occurs due to the scattering of light from inner retinal layers hence decreasing contrast of the angiographic image (4). And by the same reason, FA also has limited ability to visualize choroidal vasculature. Furthermore, the potential adverse reaction to fluorescein cannot be ignored (5, 6). OCTA on the other hand, provides depth-resolved visualization of retinal microvascular changes. At least 4 plexus can be identified using OCTA: the superficial capillary plexus (SCP), intermediate capillary plexus (ICP), deep capillary plexus (DCP), and radial peripapillary capillary plexus (3, 7).

OCTA has been widely studied in retinal vascular disorders such as diabetic retinopathy, retinal vascular occlusions and age-related macular degeneration (8, 9). In this series, authors have applied OCTA to several other retinal and optic nerve disorders resulting from developmental, inflammatory, metabolic, and other etiologies. Increasingly sophisticated image processing techniques further increase the ability to extract information from these images. Each of these work help further the current understanding of pathophysiology in these conditions.

Fragiotta et al. studied adaptive vascular arrangements in idiopathic fovea plana with volume-rendered OCTA. By means of volume rendering reconstructions, the authors demonstrated that in normal eyes, interactions between the macular microcirculation at the level of the SCP and ICP occurred more frequently compared to that between the ICP and DCP. At the parafoveal region, direct connections between the SCP and DCP were rarely seen. In contrast, eyes with fovea plana had an increase in direct connections between the SCP and DCP. It was thus proposed that the anatomical absence of foveal depression and the loss of trilaminar vascular flow resulted in an alternative vascular arrangement with interconnections between the SCP and DCP.

Bacherini et al. studied OCTA in Fabry disease (FD) and reported significantly lower vascular density in both SCP and DCP, compared to healthy controls as a result of retinal vascular abnormalities due to metabolite deposition in FD. While most published studies evaluated OCTA parameters related to vascular density, the authors also investigated vascular perfusion parameters. However, there were no significant differences in vascular perfusion indices for both the SCP and DCP comparing FD patients and healthy controls. The authors have postulated other vascular compensatory mechanisms counteracting reduced vascular density as a cause of the discrepancy. Systemic parameters such as maximal left ventricular wall thickness and glomerular filtration rate have been considered representative of cardiovascular and renal impairment in FD. Bacherini et al. analyzed the correlations between the OCTA results and the two systemic parameters but also did not find a relationship between the variables.

These studies highlight the potential for detailed, quantitative evaluation of the retinal vascular involvement with OCTA, which was not previously possible. With adequate validation, these OCTA parameters may eventually be useful as biomarkers for serial monitoring of disease progression. The non-invasiveness, ease of acquisition and relative low cost compared to neuroimaging are some of the key advantages for developing this area.

Wei et al. reported microvascular damage in the superficial plexus in neuromyelitis optical spectrum disorders (NMOSD), but these macular microvascular alterations appear to arise independently of the occurrence of optic neuritis (ON) in NMOSD. Microvascular damage in the superficial plexus was seen in eyes of patients with NMOSD with or without the presence of optic neuritis, as compared to healthy controls. Microvascular damage in the deep capillary plexus, however, was markedly present in eyes of patients with ON, and mildly present in eyes with NMOSD without optic neuritis. This suggests that primary retinal vasculopathy may exist in NMOSD (10). A recent study provided structural and functional evidence of Müller glial dysfunction in eyes of patients with AQP4-ab-positive NMOSD (11). Müller cells are possibly the target of direct attack by AQP4-ab in NMOSD, and this may account for the decrease in vessel density that is independent of optic neuritis.

Yan et al. studied peripapillary superficial microvasculature in optic disc drusen (ODD) and found that eyes with ODD had reduced vascularity. In their quadrant analysis, significant reduction in at least four out of six OCTA measurements was seen in the nasal and superior quadrants, which correspond to where ODD is most commonly found. They also found that a decrease in peripapillary OCT retinal nerve fiber layer thickness and OCTA vessel density can occur without a corresponding decline in visual function—measured by mean deviation in static perimetry. This suggests that structural changes on OCTA can predate visual field changes and may offer a more sensitive and objective diagnostic method.

These works highlight the new opportunities of evaluating retinal microvascular changes in other neurological conditions, which include dementia and Alzheimer's disease, anterior ischemic optic neuropathy, demyelinating diseases such as multiple sclerosis and NMOSD, and hereditary optic neuropathies such as Leber hereditary optic neuropathy and dominant optic atrophy. These findings will aid in enhancing our current understanding of the pathogenesis of many conditions. Neurons and retinal vasculature are closely associated due to neurovascular coupling. As such, evaluating changes in OCTA may be important in neurological disorders, even if the primary etiology is not thought to be vascular or ischemic in origin.

To enable the application of OCTA to clinical settings, further development and advances in image processing are needed, such as improved automated segmentation accuracy, and the development of standardized in-built vessel density calculation software. Concurrently, studies to establish normative values are needed. These data will potentially allow the automation of OCTA as a diagnostic tool. The study of longitudinal OCTA data in diseases can further shed light on its usefulness as a monitoring tool. Correlation between structural and functional changes and the temporal sequence of each will further direct the clinical application of OCTA, whether in the setting of screening, diagnosis or monitoring of progression. And finally, uncovering its functional correlation can empower it as a prognostic tool.

## Author Contributions

CY and SS: drafting and critical review. CC: conceptualization and critical review. All authors contributed to the article and approved the submitted version.

## Funding

This study was supported by National Medical Research Council Open Fund Large Collaborative Grant: NMRC/LCG/2008/004.

## Conflict of Interest

The authors declare that the research was conducted in the absence of any commercial or financial relationships that could be construed as a potential conflict of interest.

## Publisher's Note

All claims expressed in this article are solely those of the authors and do not necessarily represent those of their affiliated organizations, or those of the publisher, the editors and the reviewers. Any product that may be evaluated in this article, or claim that may be made by its manufacturer, is not guaranteed or endorsed by the publisher.
